# Assessment of Suitable Habitat of the Demoiselle Crane (*Anthropoides virgo*) in the Wake of Climate Change: A Study of Its Wintering Refugees in Pakistan

**DOI:** 10.3390/ani14101453

**Published:** 2024-05-13

**Authors:** Tauheed Ullah Khan, Inam Ullah, Yiming Hu, Jianchao Liang, Shahid Ahmad, James Kehinde Omifolaji, Huijian Hu

**Affiliations:** 1 Guangdong Key Laboratory of Animal Conservation and Resource Utilization, Guangdong Public Laboratory of Wild Animal Conservation and Utilization, Institute of Zoology, Guangdong Academy of Sciences, Guangzhou 510260, Chinaomifolajitk@yahoo.com (J.K.O.); 2 Institute of Biological Sciences, Gomal University, Dera Ismail Khan 29220, Pakistan; inamullah@nefu.edu.cn; 3 College of Wildlife and Protected Areas, Northeast Forestry University, No. 26, Hexing Road, Harbin 150040, China; 4 School of Ecology and Environment, Hainan University, Haikou 570228, China; 5 Center for Eco-Environment Restoration Engineering of Hainan Province, Hainan University, Haikou 570228, China

**Keywords:** climate change, migratory routs, stopovers, species habitat, KP, Baluchistan, Pakistan

## Abstract

**Simple Summary:**

Climate change and global warming have effects on every ecosystem and have caused huge losses in global biodiversity. This study identified that currently, the study area explored in this work provides a good quantity (around 35%) of suitable habitat for the Demoiselle Crane. The most influential factors determining Demoiselle Crane habitat suitability included the temperature seasonality, annual mean temperature, terrain ruggedness index, and human population density as significant contributors. Under changing climate scenarios, the study predicted a major loss of species current suitable habitat, with shrinkage and movement towards western–central areas along the Pakistan–Afghanistan boarder.

**Abstract:**

The inevitable impacts of climate change have reverberated across ecosystems and caused substantial global biodiversity loss. Climate-induced habitat loss has contributed to range shifts at both species and community levels. Given the importance of identifying suitable habitats for at-risk species, it is imperative to assess potential current and future distributions, and to understand influential environmental factors. Like many species, the Demoiselle crane is not immune to climatic pressures. Khyber Pakhtunkhwa and Balochistan provinces in Pakistan are known wintering grounds for this species. Given that Pakistan is among the top five countries facing devastating effects of climate change, this study sought to conduct species distribution modeling under climate change using data collected during 4 years of field surveys. We developed a Maximum Entropy distribution model to predict the current and projected future distribution of the species across the study area. Future habitat projections for 2050 and 2070 were carried out using two representative concentration pathways (RCP 4.5 and RCP 8.5) under three global circulation models, including HADGEM2-AO, BCC-CSM1-1, and CCSM4. The most influential factors shaping Demoiselle Crane habitat suitability included the temperature seasonality, annual mean temperature, terrain ruggedness index, and human population density, all of which contributed significantly to the suitability (81.3%). The model identified 35% of the study area as moderately suitable (134,068 km^2^) and highly suitable (27,911 km^2^) habitat for the species under current climatic conditions. Under changing climate scenarios, our model predicted a major loss of the species’ current suitable habitat, with shrinkage and shift towards western–central areas along the Pakistan–Afghanistan boarder. The RCP 8.5, which is the extreme climate change scenario, portrays particularly severe consequences, with habitat losses reaching 65% in 2050 and 85% in 2070. This comprehensive study provides useful insights into the Demoiselle Crane habitat’s current and future dynamics in Pakistan.

## 1. Introduction

The effects of climate change have reverberated across ecosystems [1,2,3,4] and caused significant global biodiversity loss [4,5,6]. Climate change and environmental conditions have profound impacts on species’ habitat [7,8], niches, and future prospects at various geographical scales [9,10,11,12,13]. Such distributional changes can challenge the survival of a wide array of life-forms, including plants [14], insects, [15,16,17], and animals [18] living in a range of habitats, from deep ocean waters to lofty mountains. Climate-induced habitat loss has not only exacerbated local and global biodiversity declines [4,19] but has also affected species population, community structure, and reproduction [20,21,22,23,24,25,26,27,28]. Habitat degradation and loss is one of the main reasons for species population declines and ultimate extinctions [29,30,31,32]. Climate change has reduced the available habitat and distribution ranges of a large number of species [20,21,22,33,34] and has left species with less favorable habitats [34], compromising their survival [4,30,35,36,37,38,39].

Like many species, migratory bird species are also affected by climate change in many different ways [40]. In the last few decades, the populations and numbers of the migratory birds have drastically declined due to climate change [18,41,42,43]. This has altered the activity pattern [44], habitat, and distribution range of avian species [45]. Changes in climate could also change the amount of time these species spend at their breeding and wintering grounds [46]. The migratory routes of these species are also affected by global climate change [47]. This situation could increase the competition between the native and migratory birds in terms of accessing the limited available food resources. Changes in climate variables have impacts on the demography, growth, population, and reproduction of these species [48].

**Figure 1:**
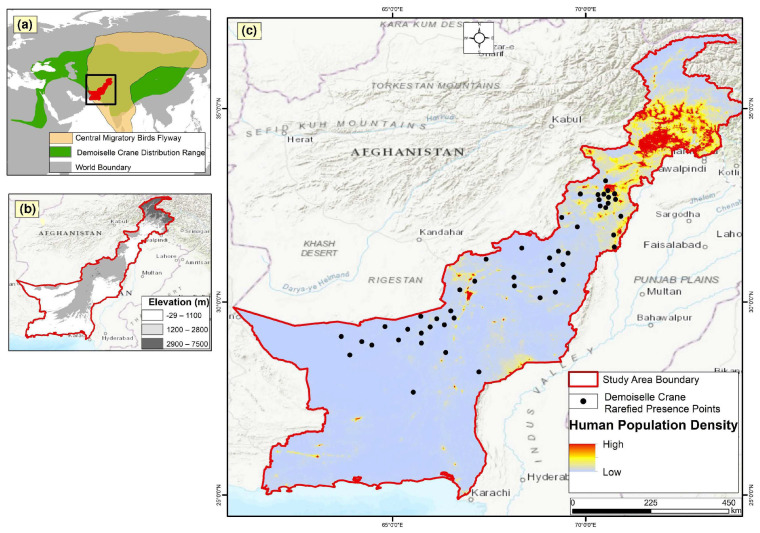
Location of study area with respect to crane distribution range and the migratory bird’s central flyway (**a**), study area location, elevation range (**b**), and human density (**c**). The black dots show the rarified presence points of the species obtained during the field surveys.

The Demoiselle Crane (*Anthropoides virgo*), with a global population estimated to exceed 230,000–261,000 individuals, is a migratory bird species with a widespread distribution [49]. It undertakes a perilous journey between breeding grounds in the European territories [50,51] and the Asian segment [50] and through the Indus Flyway, seeking winter refuge in Pakistan [52]. Despite its negligible contributions to carbon global emissions, Pakistan is ranked the fifth most vulnerable country in the world and is often affected by extreme climatic calamities and weather events [53,54]. In Pakistan, climate change is causing a rise in the average annual temperature, unprecedented rainfall, glaciers shrinkage, and frequent flood events; these issues are damaging many ecosystems across the country [55,56,57,58,59,60], and all contribute to the country’s loss of biodiversity and species-suitable habitats [61,62]. In Pakistan, different climatic threats and anthropogenic pressures have caused the immense degradation of stopovers and habitat of migratory species [63]. Demoiselle Cranes are one such example. In Pakistan, this species flies through the Balochistan and Khyber Pakhtunkhwa provinces (Figure 1a–c) along different valleys including, Zhob, Gambilla, Kurram, Kech, and River Indus [64]. This migration route is fraught with significant challenges, including illegal hunting pressures, physical obstacles, adverse weather conditions, predatory threats, food scarcity, wetland habitat depletion, and exposure to pesticides [52,64,65,66,67].

The Demoiselle Crane’s native breeding grounds become highly unhospitable for the species in winter due to harsh weather and food scarcity. Alternatively, Pakistan maintains an ideal winter temperature for the species, making it a site of winter refuge [52,68], as the mean winter temperature in Pakistan ranges between 18 and 20 °C. The species visits our study area (Khyber Pakhtunkhwa and Baluchistan) during the winter and spring seasons. However, a rise in the annual average temperature has been registered for the country. Moreover, a rise of 3 to 5 °C in the mean annual temperature of the country is expected to occur by the end of this century [69]. This study is an attempt to assess how increases in temperature and associated environmental anomalies will affect the habitat suitability of Demoiselle Crane in Pakistan. We hypothesized that as temperature rise and environmental conditions shift in Pakistan as a result of climate change, the habitat suitably for the Demoiselle Crane will decline. This study uses the Maximum Entropy (MaxEnt) model to assess the current and future habitat and distribution of Demoiselle Crane in Pakistan.

## 2. Materials and Methods

### 2.1. Study Area and Our Species of Interest

Pakistan has a very diverse geography ranging from the Arabian sea in the south to the second-highest mountain peak (K-2) in the world in the north [70]. The country’s diverse geography provides refuge for a large number of resident and migratory bird species [71]. Pakistan provides passageways and flying routes for many migratory birds, which collectively and commonly pass through the Indus Flyway (Figure 1a) [52]. The Indus Flyway extends from Karakoram Mountain Range to the Indus River Delta in the south of the country. The study area lies between 61° and 75° East and 24° and 37° North, over an area of 458,383 km^2^ (Figure 1a–c) and is identified as an Important Bird Area (Birdlife International, 2022). It extends from the coast of the Indian Sea in the south to the Karakorum, Hindu Kush, and Himalayan Mountain ranges, attaining an elevation of 7289 m in the north (Figure 1b) [72]. The diverse geography of the study area supports a rich assemblage of biodiversity. Siting on the Indus flyway zone and situated on the central flyway of migratory birds (Figure 1a), the area plays a crucial role as a stopover for migratory birds, especially during the winter season [73]. The central flyway is a vital thoroughfare for numerous migratory bird species, especially cranes seeking winter sanctuaries and stopovers in Pakistan [64,66,74,75,76,77]. The Demoiselle Crane migrates to and from wintering grounds over the Himalayas and the Hindu Kush mountains, with stopovers at key waypoints in the study area [77]. This species is more prevalent in the area during their autumn and spring migrations [67,78,79]. Demoiselle Crane distribution modeling was carried out in the Khyber Pakhtunkhwa (KP) and Balochistan (BA) provinces, which are known wintering grounds for the species in Pakistan [67,76]. 

### 2.2. Occurrence/Presence Data

Field surveys were carried out over four years between 2018 and 2022 across diverse habitats and environmental conditions in the study area to gather data on the presence and distribution of the Demoiselle Crane. We could not carry out the surveys in 2020 due to COVID-19 [80] restrictions, as movement was limited and visits were not possible. The questionnaire surveys and interviews were administered in tandem with field visits with a diverse set of respondents, including wildlife experts, local inhabitants, crane keepers, and seasonal hunters, to obtain specific input from local communities living in our study area. We obtained the formal consent of the respondents before we began to fill out the questionnaire. The respondents were informed about the purpose of the surveys, and that the collected data will be used solely for research purposes and will not be used to harm any person or community in any way. Additionally, they were informed that all personal details will remain confidential and will not be disclosed to any third parties. Moreover, the volunteers were informed that they had the right to withdraw from the survey at any moment if they felt uncomfortable. After that, before the interview formally began, photographs were utilized to help respondents to identify the Demoiselle Crane. The questionnaire included questions on Demoiselle Crane status, the estimated population in captivity in the area, how many they personally own, their distribution, historical range, hunting area, best hunting season, and last sighting. In addition, the interviewers’ conversation was recorded on audio tape, and any questions were answered soon after the interview was completed. The records were then compared to data from field surveys. These interviews aimed to elicit information on the species’ presence, its potential habitat areas, and locations frequented for crane hunting and live capturing [65]. Questionnaire surveys are among the most reliable source of information about species and species status in a particular area [81,82,83].

The study also incorporated an examination of published literature on different ecological aspects of Demoiselle Crane in Pakistan, as previous literature provides a reliable amount of information about the species’ occurrence and distribution [84]. Numerous studies have used presence data from the published literature for species distribution modeling [85,86,87]. Google Earth (http://ditu.google.cn/, accessed on 16 January 2024) was used to ascertain coordinates for presence points extracted from the literature (Table S1). For analysis, we prioritized using the presence points collected during the field surveys due to their accuracy and reliability. For instance, where we had presence points from three sources, including field surveys, questionnaire interviews, and the literature, we cross-refenced and selected the field survey points. This meticulous approach allowed us to ensure the quality and validity of our data while leveraging insights from various resources to enrich our study.

### 2.3. Preprocessing of Occurrence/Presence Data

The presence points were converted into a point shapefile using ArcGIS 10.5 (Esri, Redlands, CA, USA). To minimize the effect of redundancy on the model prediction results, duplicate points and outliers were removed, and spatially autocorrelated points were excluded using the ArcGIS SDM Toolbox [87,88,89,90]. Finally, coordinates (projection WGS 1984) of the rarefied occurrence points were used in the MaxEnt model.

### 2.4. Procurement, and Pretreatment of Predictor Variables

In species habitat suitability modeling studies, environmental variables, including temperature, rainfall, geographical features, and geological attributes, were meticulously selected due to their significance in shaping species distributions [91]. WorldClim Bioclimatic variables could reflect the properties of climatic and seasonal variation [91]. Elevation and nineteen bioclimatic variables were obtained from the WorldClim database (www.worldclim.org, accessed on 18 February 2024) for the current period and for future scenarios in 2050 and 2070, with a 2.5 arc minute spatial resolution. These variables play crucial role in defining species habitat niches [92,93], and are widely employed in the field of species distribution modeling [36,91,93,94]. In addition to these, other variables, including Pakistan’s human population (pop), land use land cover of the area (lulc), soil, normalized difference vegetation index (NDVI), roads density, and terrain ruggedness index (rugged) were included for MaxEnt modeling. The bioclimatic variables were used as a baseline for the projection of species habitat under different future climatic scenarios [95,96]. Details and sources of the parameters used for species modeling are provided (Table S2).

### 2.5. Preprocessing of Current Climatic Data

For the initial model, a set of twenty-eight environmental variables was considered. To ensure data consistency, variables were standardized and projected to a unified resolution and coordinate system. In accordance with Worthington’s study [96], variables that exhibited low contribution percentages (a percentage contribution of 0.25 or 0) in the Jackknife test were eliminated to reduce model overfitting [96,97,98]. As many bioclimatic variables are spatially correlated, the remaining environmental variables were also tested for collinearity, as highly correlated variables could also lead to model overfitting and inaccurate results [98,99]. A Pearson Correlation Coefficient test with a threshold of |r| ≥ 0.75 [100] (Figure S1) was applied to identify and remove highly correlated variables to further improve the model simulation accuracy. Ultimately, eight environmental variables with low correlation were retained and used for the species habitat suitability modeling [100]. These variables included annual mean temperature, temperature seasonality standard deviation, precipitation of the wettest quarter, precipitation of the coldest quarter, human population, land cover, and terrain ruggedness index (Table 1). With the exception of the BioClim variables, the remaining chosen variables remained unchanged for the projection of habitat suitability in the future.

### 2.6. Future Projection Data

For future climate modeling, this study used two representative concentration pathways (RCP 4.5, and RCP 8.5) (IPCC, 2014) [101]. These RCPs represent future scenarios, considering the amount of greenhouse gases (GHGs) that will be emitted in the future. Future climate scenarios were established for the GHG emissions scenarios [102] for the years 2050 (the average for 2031–2050) and 2070 (the average for 2061–2080). For future projection, the bioclimatic variables were derived from a global circulation model (GCM) including HadGEM2-CC, BCC-CSM1-1 and CCSM4. BCC-CSM1-1 was used for the period of 2050 and 2070 for all the mentioned RCPs. The HadGEM2-CC (Hadley Global Environment Model 2 Carbon Cycle) was developed by the Hadley Center, United Kingdom [103]. HadGEM2 models have been used to perform all the CMIP5 (Coupled Model Intercomparison Project Phase 5) centennial experiments, including ensembles of simulations of the RCPs. HadGEM2-CC is one of the models that was used by the international governmental panel on climate change (IPCC) in its fifth Assessment Report (AR5). BCC-CSM1.1 is among the most-used models currently available for simulating the global climate response to increasing greenhouse gas concentrations. Our four selected future climate data sets were downloaded from the World Climate Database [104]. The CCSM4 (The Community Climate System Model v. 4.0) developed by the National Center for Atmospheric Research (Colorado, United States) from the WorldClim database under both scenarios over the periods 2050 (average for 2041–2060) and 2070 (average of 2061–2080). CCSM4 is an efficient global climate tool for the simulation of future climatic conditions, which has been thoroughly evaluated in the region and successfully applied to predict the influence of future climatic changes on the distribution of plant species in similar environments [105]. Researchers have successfully used these models in the study region [106,107,108].

### 2.7. Construction of Maximum Entropy (MaxEnt) Model

This study used a free, open-source, and Java-based MaxEnt (https://biodiversityinformatics.amnh.org/open_source/maxent/, accessed on 25 October 2023), software (v 3.4.3) [109,110] to identify, categorize and quantify the suitable habitat available for Demoiselle Crane in the study area. It is an efficient model [111,112,113,114] which uses the known geographical distribution of a species and associated climatic and geographical data to calculate the potential distribution of a species in a given area [62,99,102,113]. Moreover, it is a better choice to use when occurrence data include only presence points [99,115,116,117,118,119,120] and no absence data, which are difficult to obtain with accuracy [88,99,113,121]. The occurrence and predictor variables data (unified projection and resolution) were added to the MaxEnt software. Next, the MaxEnt model parameters were calibrated. Depending on the circumstances of each specific study, data and species model parameters could be set in many ways [122,123,124,125]. For this study, we used cross-validation method with 5000 iterations and 10,000 background points. We ran ten replicates to determine the average probability for a habitat suitability map of the species [126]. The rest of the parameters retained their default settings. These model calibrations improved the accuracy and prediction of the MaxEnt model [127]. Finally, to evaluate the fitness and accuracy of our final MaxEnt model, we used the area under the curve (AUC) [128]. The AUC values for a model range from 0 to 1. AUC values closer to 1 demonstrate that the model has more accurate outputs [21,99,129,130,131]. The Jackknife test was used to determine the percent contribution and relative importance of predictor variables for Demoiselle Crane habitat suitability and distribution [113,132,133].

### 2.8. Division of Potential Suitable Growing Areas for Demoiselle Crane

The model simulation results (raster layer) were reclassified using the Jenks natural breaks classification method in GIS. This method reclassifies the raster based on the fact that the differences within each category/class are as small as possible, while the differences between categories/classes are as large as possible. This study reclassified the potential species habitat into four classes, including unsuitable habitat (≤0.10), less suitable habitat (0.11–0.30), moderately suitable habitat (0.31–0.70), and highly suitable habitat (≥0.71), based on several studies [14,36,92].

## 3. Results

### 3.1. MaxEnt Model Prediction Evaluation

**Figure 2:**
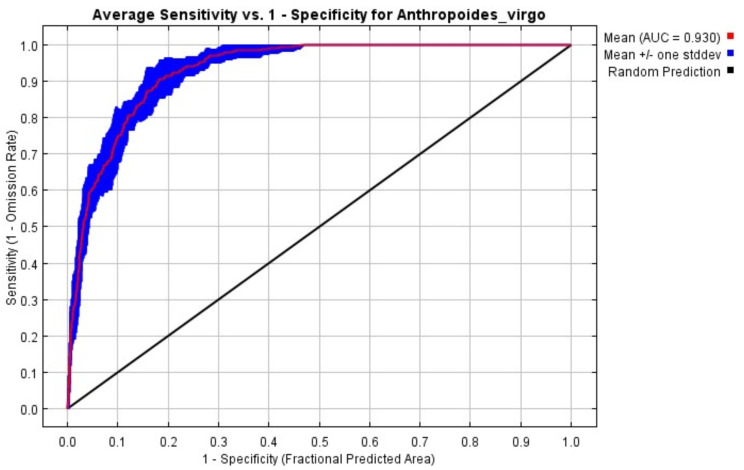
AUC test results of MaxEnt modeling carried out to assess the habitat suitability of the Demoiselle Crane.

We collected a total of 153 occurrence/presence points for the Demoiselle Crane from the study area. After removing spatial autocorrelation (each point at 5 km distance), we obtained 63 presence points (Figure 1d) for the Demoiselle Crane for MaxEnt habitat modeling. To evaluate the fitness and accuracy of our final MaxEnt model, we used the receiver operating characteristic (ROC) and the area under the curve (AUC). Our MaxEnt analysis obtained a valid and robust model, as indicated by the resulting AUC value. The ROC results exhibited an average AUC of 0.930 for replicate runs and a minimal standard deviation value of 0.013 (Figure 2), signifying that the model’s suitability was excellent.

### 3.2. Predictor Variables Defining the Habitat Suitability of Demoiselle Crane

The contribution of each variable in defining and predicting Demoiselle Crane distribution and habitat selection based on the MaxEnt model is given in (Table 1). Our model results show that the temperature seasonality (standard deviation × 100), annual mean temperature, terrain ruggedness index, and human population density played significant roles (with cumulative 81.3% contribution) in shaping and defining the distribution of the Demoiselle Crane in the study region under the current projection scenario. Details of the least contributing predictors are given in (Table 1). The MaxEnt model generated response curves for our eight predictor variables. These response curves exhibit correlation between species habitat suitability and individual variables by plotting habitat suitability against the values of each variable. They also give insight into the variable thresholds for species presence. A graphical representation of the response curves of the top four contributing predictor variables in this study are provided (Figure S2). The Jackknife test results demonstrated that the temperature seasonality was the predictor variable with the highest gain when used in isolation (Figure S3). This demonstrates that temperature seasonality provides the most useful information when considered on its own. Moreover, omission of this variable caused a decrease in the gain. Therefore, temperature seasonality appears to have the most information that is not present in the other predictor variables.

**Figure 3:**
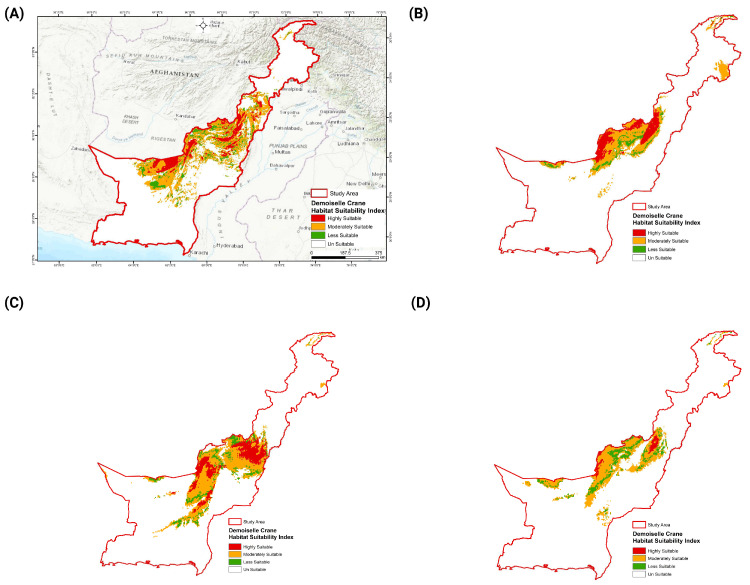
The distribution of suitable habitats of Demoiselle Crane across study area. Plate (**A**) represents the current suitable habitat, while projected habitat changes and shift under future climatic scenarios are exhibited by (**B**) BCC-CSM1-1, (**C**) HADGEM2-AO, and (**D**) CCSM4.

**Figure 4:**
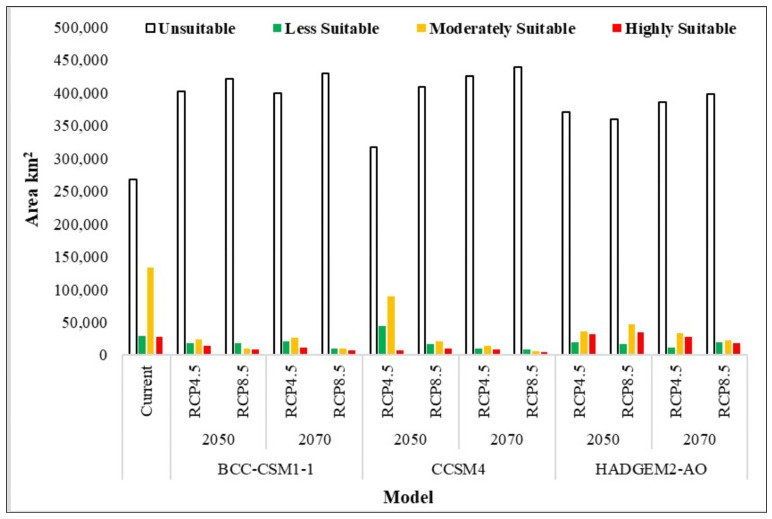
Categorization and quantification of Demoiselle Crane habitat (km^2^) under current and future climate change.

Our model predicted the highly suitable habitat of the species over an area of about 27,911 km^2^. The highly suitable habitat of the Demoiselle Crane predicted by the model was primarily distributed in the southern districts (Lakki Marwat, Tank, Southern Waziristan, Bannu, and Karak) of KP province, while a major proportion of highly suitable habitat was also predicted in some districts of Baluchistan province: Zhob, Mastung, Quetta, Musakhel, Ziarat, Harnai, Loralai, Nushki, Pishin, and Qilla Saifullah. A small chunk of suitable habitat was also predicted in the eastern part of the Chitral district located in the northern part of our study area (Figure 3). The moderately suitable habitat of the species was found over an area of about 134,068 km^2^ (Table S3) and was mostly concentrated in the central part of the study area, northern and central parts of Baluchistan province, and the southern range of KP. The less suitable habitat of the species was predicted over an area of approximately 28,865 km^2^ and fell close to moderately suitable habitat spanning an area of about 134,068 km^2^, while the unsuitable habitat, covering 267,539 km^2^ (Figure 4, Table S3), was found in the extreme southern and northern parts of our study area (Figure 3).

### 3.3. Future Distribution of Demoiselle Crane Habitat

Our projection models predicted habitat change and movement for the Demoiselle Crane in the study area (Table 2, Figure 3). BCC-CSM1-1 future model projection showed that under the RCP 4.6 and RCP 8.5 scenarios, the species could lose 51%, and 67% of its currently highly suitable habitats to climate change in 2050, respectively. The model predicted a suitable habitat loss of 60% and 72% under RCP 4.6 and RCP 8.5 in 2070, respectively (Table 2). Under the RCP 4.5 CCSM4 scenarios for 2050 and 2070, the Demoiselle Crane lost 75% and 71% of highly suitable habitats, respectively. In the extreme climate change scenario of RCP 8.5 in 2050, the Demoiselle Crane lost 65% of its currently highly suitable habitats, while in the extreme scenario of RCP 8.5 in 2070, the species’ currently suitable habitats shrunk by 85% (Table 2). Interestingly, the currently suitable habitats of the Demoiselle Crane increased under different RCPs using the HADGEM2-AO climate model for 2050, but again showed declines of 1% and 37% under the modeled RCPs by 2070. The species tends to retain a small suitable habitat in the upper Chitral area in all future scenarios.

### 3.4. Movement of the Demoiselle Crane’s Suitable Habitat

In general, under all climatic scenarios of all the three models that were used, we observed shrinkage and a shift of the suitable habitat towards the western–central parts of our study area (Figure 3B–D). This observed shift was concentrated in the southern districts of KP, including Lakki Marwat, Tank, and South Waziristan, and the adjacent districts of Baluchistan, including Zhob, Pishin, Loralai, Noshki and Dalbadin. These areas lie near the Pakistan–Afghanistan border.

## 4. Discussion

We carried out species distribution and habitat suitability modeling to categorize and quantify suitable habitat for Demoiselle Crane in two provinces in Pakistan bordering Afghanistan. Habitat selection was investigated using a MaxEnt modeling approach, which allowed for the assessment of environmental variable contribution to species distribution. In this study, the MaxEnt model showed its accuracy and efficiency, as the model achieved an AUC value greater than 0.9, and thus is considered an excellent prediction model [74,134,135,136,137].

The MaxEnt model highlighted the importance of temperature seasonality, annual mean temperature, the terrain ruggedness index of the area, and human population density in shaping the distribution of Demoiselle Cranes. These findings align with previous studies emphasizing the role of climatic variables and landscape features in determining species distribution [138,139,140]. The response curves of contributing factors exhibited a useful understanding of the species response to climatic variation and ecological preferences. They also give insight into the correlation between species habitat suitability and presence and the used predictor variables. For instance, the negative correlation between habitat suitability and human population density reflects the species’ aversion to areas with higher human disturbance, aligning with the general understanding of human–wildlife interactions [141]. Moreover, distinct threshold effects were observed in response to all selected variables. The sudden rise and decline in habitat suitability beyond a particular value indicates a critical variable threshold. This highlights the non-linear nature of species-environment relationships and underscores the importance of considering threshold effects in conservation planning.

Mean annual and seasonal temperature can influence the migratory, foraging behavior, and site selection of a species [142,143,144]. Our results indicated that temperature seasonality and annual mean temperature play major roles in defining Demoiselle Crane habitat suitability. The mean winter temperature in Pakistan ranges between 18 and 20 °C. Demoiselle Crane breeding grounds are distributed from European Russia into Kyrgyzstan, China, and Mongolia [50,145]. However, their native breeding ground can become highly unhospitable for the species in winter due to harsh weather and food scarcity. Alternatively, Pakistan maintains an ideal winter temperature for the species, making it a site of refuge [52,68]. The species visits our study area during the winter and spring seasons. Therefore, seasonal temperature and mean annual temperature played a substantial role in defining distribution and habitat in this region. The effect of annual mean temperature and temperature seasonality has been reported as shaping the site use and habitat suitability of other crane species as well [138].

The significance of the terrain ruggedness index in habitat selection is consistent with previous literature, as cranes often prefer areas with wide-ranging topography and open grounds for resting, foraging, and roosting [138]. In our results, the Demoiselle Crane showed the highest preference for areas with low ruggedness. The species prefers to stay in open and plain lands because such areas typically provide grasses, grains, and insects, which comprise the preferred diet of the species in this region [146]. Other long-necked crane species prefer a similar diet [147]. Additionally, crane species prefer to rest in areas with open ground and bountiful food resources. Therefore, the species tends to land in open pastures, croplands, marshes, and river beds [148,149]. During our field visits and questionnaire surveys, it was observed that the hunters used to build their hunting facilities and huts in or near open grounds in the study area. According to the hunters, large and flat open grounds are the best location for hunting, as the species prefers to land and rest in such locations. The species also prefers areas with open grasslands and cultivated farmlands [138], so long as the human disturbance is minimal. Pasture and croplands also provide their optimal dietary resources, including grasses, grains, and insects.

In our results, human population density emerged as a significant factor in defining habitat suitability and site selection, indicating the high impact of anthropogenic activities on crane distribution, possibly through disturbance or habitat alteration. The habitat suitability was reported to be lowest in areas with the highest human density. We believe that this species tends to avoid densely populated areas to avoid illegal hunting, which is very common in the area [55,150]. While hunters typically target the species in open-ground areas, they are indiscriminate in pursuing the species if the cranes are seen flying overhead, even if the birds are out of firing range (personal experience of the first author). Such disturbances can divert the flock from its migratory path. The species may fly very high over populated areas [151] or at night, as human density and disturbance can alter the route and resting stages of the species [141,152]. The future projection of Demoiselle Crane distribution and habitat, under different RCPs and climatic models, presented a consistent trend of habitat loss, indicating the vulnerability of the species to climate-induced alterations in environmental conditions. The extreme climate change scenario (RCP 8.5) portrays particularly severe consequences, with habitat losses reaching 65% in 2050 and a staggering 85% in 2070. This highlights the urgency of addressing climate change adaptations and mitigations measures to safeguard the Demoiselle Crane’s habitat. Such drastic reductions in suitable habitat may have cascading effects on species population dynamics and ecological interactions.

**Figure 5:**
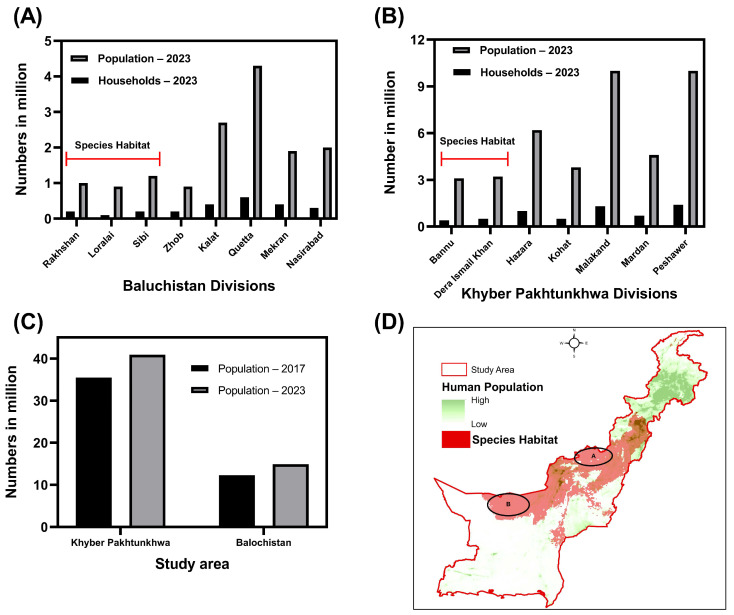
Spatial distribution of human population and species habitat in the study area. Plates (**A**) and (**B**) represent the current human population and household numbers in administrative units in BAL and KP, respectively. Plate (**C**) shows the population changes in BAL and KP in 2017 and 2023 according to the national population census. Plate (**D**) exhibits the spatial distribution of the Demoiselle Crane’s suitable habitat and the human density, and location of proposed protected areas (A and B: black ellipse circle).

Pakistan is among the top five countries facing devastating effects of climate change. Pakistan has experienced devastating flood events, severe heatwaves, and irregular as well as unexpected heavy rainfall in recent years. These extreme environmental anomalies are expected to increase in future if the current trend continues. Our future projections showed that the species is expected to lose a major proportion of its current habitat (which is considered moderately and highly suitable as a species habitat). This loss could be attributed to the ongoing climatic anomalies and ever-increasing human population of Pakistan. Currently, the areas offering suitable habitat to the species are those with plain open grounds and a relatively lower human population density (Figure 5A,B,D). There are more suitable grounds spanning an area of (5313 km^2^) with a relatively lower human population density in Khyber Pakhtunkhwa compared to northern districts, including Peshawar, Mardan, Charsadda, Hazara, and Abbottabad. Moreover, compared to the northern region, this part of the province receives less [153], but sufficient, rain to support natural vegetation growth and natural wetlands. Notably, the observed shift in suitable habitat towards the western–central parts of the study area, particularly in the southern districts of Khyber Pakhtunkhwa and adjacent districts of Baluchistan, signals a spatial reconfiguration of suitable conditions for the Demoiselle Crane. These areas near the Pakistan–Afghanistan border exhibit a concentrated shift in suitable habitat. We presume that these areas have lower human populations, and therefore the related consequences for the environment and habitat would be minimal (Figure 5D).

Human presence, disturbance, and infrastructure play key roles in species resource selection. Demoiselle Cranes appear to prefer areas for resting, breeding, and foraging that have minimal human presence and disturbance [154]. Distance from the food resources [155,156,157,158,159,160], human disturbance, and associated predation risk determine a species’ habitat selection [159,161]. Human presence also affects the foraging grounds of a species both spatially and temporally [161,162,163]. Thus, all factors must be taken into consideration to understand a species’ distribution and habitat requirements [84].

The observed decrease in suitable habitat could be attributed to Pakistan’s rapidly growing population. Pakistan is the fifth most populous country in the world, with a population of 210 million people [https://www.unodc.org/pakistan/en/country-profile-pakistan.html, accessed on 25 October 2023], and a current average annual population growth rate of 2.3% and rising (Supplementary Materials, Figure S6). Subsequently, settlements and associated infrastructures are expanding into species’ core habitats. According to 2023 human population census data, the KP population (density: 300 people/km^2^) has increased by 15.09%, from 35.5 million in 2017 to 40.86 million in 2023 (Figure 5C). The Balochistan population (density: 143 people/km^2^) has ballooned by 56.10% from 2017 (12.34 million) to 2023 (19.26 million) (Figure 5C).

The impacts of climate change in Pakistan are far worse than in other countries in the region. Pakistan is fifth on the list of countries that are most vulnerable to natural calamities and climate change [53]. Currently, very little is known about how severely climate change will affect the diverse and fragile ecosystems and weak economy of the country [164]. A study projected a rise of 3.8 °C in the mean temperature of the country by 2100 [165]. Another study forecasted increases of 13.7 °C and 6.0 °C in the average annual temperature of the country by 2060 and 2090, respectively [166]. In the last five decades, an 0.5 °C rise in annual mean temperature of the country has been noted [69]. Species like the Demoiselle Crane that seek winter refuge in the country will likely face more dire consequences due to the projected rise in temperature [166].

Given the potentially dire consequences linked to future habitat degradation, we propose some conservation strategies. Firstly, regular and long-term monitoring of species-suitable habitats should be carried out. This would help us to assess the consequences connected to expected reductions and alterations in habitats, which will in turn assist with the swift identification and response to newly developing future challenges associated with conservation. In this regard, it is recommended to carry out continuous seasonal and annual field surveys across the species’ current suitable habitat. Second, to minimize habitat loss, it is recommended to establish new protected areas and expand the existing areas across this species’ habitat and, ideally, its entire distribution range throughout the country. This would help to protect the species’ habitat in the long run. The newly protected areas should be established in connection to the pre-existing protected area to strengthen the protected areas network. The management of existing protected areas also needs to be strengthened and upgraded to align with new conservation challenges. To protect the species’ habitat and control illegal hunting, the staff of the protected areas should be trained and supported logistically to carry out their duties more professionally and efficiently in this remote area. Local governments and concerned departments should protect the habitat of this species outside its protected area by declaring the wider area as a buffer zone area. This will help to minimize any agricultural or other developmental activities in the unprotected areas of the species’ habitat. Based on our results, we recommend designating protected areas (proposed protected areas A and B: black ellipse circle) in areas with highly suitable habitat and low human population densities (Figure 5D). This will help to protect habitat and minimize illegal hunting pressure.

Third, the local governments, concerned departments, and wildlife conservation NGOs should collaborate and initiate schemes and projects to educated local communities on the sustainable use of natural resources, livelihood practices, and community-based species conservation to decrease habitat degradation and the massive amounts of capturing and illegal hunting of this species. The existing literature acknowledges the role of community awareness in species conservation [68,167,168]. Fourth, government departments are recommended to strengthen forest protection and restoration practices and laws to combat the rising CO_2_ levels that cause rises in land-surface temperatures and subsequent species habitat loss. This will help to keep the temperature and overall climate of the area suitable for this species. Communities that are dependent on the forest for their domestic uses should be provided with alternative resources to reduce pressure on the forest. Fifth, the central–western part of our study area along the Pakistan–Afghanistan border retained a fair amount of suitable habitat for the Demoiselle Crane in our current and all projected future climatic models. Hence, the two countries should follow environmental protection protocols before, during, and after making large-scale trans-boundary developments.

## 5. Conclusions

This study was conducted to identify, categorize, and quantify the Demoiselle Crane’s habitat and distribution under current and future climatic scenarios in two provinces of Pakistan, namely Khyber Pakhtunkhwa and Baluchistan, using MaxEnt modeling. The model predicted that temperature seasonality, annual mean temperature, the terrain ruggedness index, and the human population density of the area were the predictor variables that contributed the most significantly to defining the species’ habitat and distribution. This species is destined to lose a massive chunk of its current suitable habitat to climate change across various climatic scenarios, highlighting its vulnerability to environmental changes. Strategies to conserve the Demoiselle Crane include regular monitoring of its habitat, expanding and establishing protected areas, engaging local communities in species conservation, and enhancing ecosystem protection to combat climate change. This study emphasizes the need for adaptive management practices that account for dynamic ecological interactions in the face of ongoing climate challenges.

## Figures and Tables

**Table 1:** Percent contribution and permutation importance of predictor variables.

Predictor Variable	Percent (%) Contribution	Permutation Importance
Temperature seasonality (standard deviation ×100)	29.6	19.1
Annual mean temperature	23.5	42.9
Ruggedness index of the area	18.1	2.6
Human population density	10.1	3.6
Precipitation of wettest quarter	7.2	2.6
Min temperature of coldest month	6.1	10.2
Precipitation of coldest quarter	3.1	7.7
Land cover	2.3	11.3

**Table 2:** Change (%) in suitable Demoiselle Crane habitat (km^2^) resulting from climate change.

Future Projection		Scenario	Habitat Categories			
Model	Year		Unsuitable	Minimally Suitable	Moderately Suitable	Highly Suitable
		Current	267,539	28,865	134,068	27,911
BCC-CSM1-1	2050	RCP4.5_Change	51	−38	−82	−51
		RCP8.5 Change	57	−37	−92	−67
	2070	RCP4.5 Change	49	−25	−81	−60
		RCP8.5 Change	61	−65	−93	−72
CCSM4	2050	RCP4.5 Change	19	52	−33	−75
		RCP8.5 Change	53	−41	−84	−65
	2070	RCP4.5 Change	59	−64	−89	−71
		RCP8.5 Change	65	−72	−95	−85
HADGEM2-AO	2050	RCP4.5 Change	39	−31	−73	12
		RCP8.5 Change	35	−41	−65	24
	2070	RCP4.5 Change	44	−59	−75	−1
		RCP8.5 Change	49	−33	−83	−37

## Data Availability

The data will be available on request.

## Supplementary Materials

The following supporting information can be downloaded at: https://www.mdpi.com/article/10.3390/ani14101453/s1, Table S1: literature studied to obtained occurrence points; Table S2: Details of the variables/predictors used in this study. The environmental variables used in modelling potential habitats for demoiselle crane are denoted in bold text; Figure S1: Correlation among the 8 variables retained based on the Jackknife test and contribution values; Figure S2: Demoiselle crane habitat suitability (selection) response curves for top four highly contributing predictor variables (Red curves (lines) exhibit the mean response of the ten replicates (runs) of MaxEnt model, while the blue shades indicate mean +/− standard deviation. The range of predictor variable is presented on the X-axis, while habitat suitability index (logistic output) is given on the Y-axis); Figure S3: Jackknife test of the regularized training gain of predictor variables tested in the demoiselle crane habitat suitability; Figure S4: Maps illustrating multivariate environmental similarity surface (MESS) approach for demoiselle cranes under the year 2050 Representative Concentration Pathway (RCP4.5) and (RCP8.5) for different Global Circulation Models. Negative values indicate novel climate in the MESS map across the range; Figure S5: Maps illustrating multivariate environmental similarity surface (MESS) approach for demoiselle cranes under the year 2050 Representative Concentration Pathway (RCP4.5) and (RCP8.5) for different Global Circulation Models. Negative values indicate novel climate in the MESS map across the range; Figure S6: Projection of human population in Pakistan (sources UN); Table S3: Categorization and quantification of Demoiselle Crane habitat (km^2^) under current and future climate change.
