# Supplementary material for: Assessment of Suitable Habitat of the Demoiselle Crane (Anthropoides virgo) in the Wake of Climate Change: A Study of Its Wintering Refugees in Pakistan
Source: Animals (Basel). 2024 May 13;14(10):1453. doi: 10.3390/ani14101453 (PMC11117222; doi:10.3390/ani14101453)
Supplement: Supplementary file 1 [file animals-14-01453-s001.zip › animals-2945256-supplementary.pdf]

Supplementary Material. Table: S1: literature studied to obtained occurrence points

S.No.	Paper Title	Year	Authors	Journal Name
1.	Pressure from hunting on crane species in southern districts of northern Pakistan.	2010	PERVEEN, F., & KHAN, H. U	Avian Research
2.	POPULATION ASSESSMENT OF MIGRATORY CRANES AT DISTRICT ZHOB, BALOCHISTAN	2011	Khan, B., Iqbal, M., & Ali, A	Pakistan Journal of Forestry
3.	Captive breeding of Demoiselle Crane in Lakki Marwat, Khyber Pakhtunkhwa, Pakistan.	2011	Mahmood, T., Amin, N., & Rais, M.	Беркут
4.	Captive breeding practices in Pakistan: A review.	2011	Ali, Z., Bibi, F., Mahel, A. Q., Firdous, F., & Zamaan, S. U	Journal of Animal and Plant Sciences
5.	Cranes in Pakistani folklore: Symbols of wisdom and longevity	2013	Malik, Z.	Cultural Anthropology
6.	Diet composition of the Demoiselle crane (Anthropoides virgo) migrating through Lakki Marwat, Pakistan.	2013	Sarwar, M., Hussain, I., Khan, A., & Anwar, M.	Avian Biology Research
7.	Diet composition of the Demoiselle crane (Anthropoides virgo) migrating through Lakki Marwat, Pakistan.	2013	Sarwar, M., Hussain, I., Khan, A., & Anwar, M.	Avian Biology Research
8.	Wetland degradation and its impact on crane populations in Pakistan	2015	Khan, A., et al.	Conservation Biology
9.	Challenges in conserving the Grey Crowned Crane in Pakistan	2018	Mahmood, S.	Wildlife Conservation
10.	Human-crane conflict and crop damage mitigation strategies in rural Pakistan	2019	Ali, R. & Qureshi, S.	Human-Wildlife Interactions
11.	Habitat restoration and community-based conservation for cranes in Pakistan	2020	Khan, S. & Ahmed, A.	Journal of Wildlife Management
12.	Wild birds trade in Dera Ismael Khan and Bannu divisions of Khyber PakhtunKhwa (KPK) Province, Pakistan.	2021	Hussain, A., & Khan, A. A	Brazilian Journal of Biology
13.	Hunting pressure on two migratory species, Common Crane (GRUS GRUS) and Demoiselle crane (Anthropoides VIrgo) in Khyber Pakhtunkhwa Province, Pakistan	2021	Tariq Ahmad1 , Faiz-ur-Rehman2 , Gul Saba1 , Nadeem Munawar1* , Tariq Mahmood1 , Faraz Akram3 and Ammara Baig	International Journal of Biosciences
14.	Major threats and habitat use status of Demoiselle crane (Anthropoides virgo), in district Bannu, Pakistan.	2021	Rehman, J. U., Alam, S., Khalil, S., Hussain, M., Iqbal, M., Khan, K. A., ... & Habiba, U.	Brazilian Journal of Biology
15.	Differences in on-ground and aloft conditions explain seasonally different migration paths in Demoiselle crane.	2022	Galtbalt, B., Batbayar, N., Sukhbaatar, T., Vorneweg, B., Heine, G., Müller, U., ... & Klaassen, M	Movement Ecology
16.	Hunting Pressure on Migratory Demoiselle Cranes in Pakistan.	2022	Sarwar, M., Hamid, A., & Hussain	Pakistan Journal of Zoology
17.	Clicks and comments: Representation of wildlife crime in Pakistan in social media posts	2023	Haq, R. U., Abdulabad, A., Asghar, S., & Szabo, J. K.	Global Ecology and Conservation,
18.	Determining the relative abundance of, habitat preferences of and occurences of gastrointestinal parasites in common crane and demoiselle crane inhabiting three distinct habitats.	2023	Ullah, I., Sun, X. Y., WU, Q. M., Deng, W. Y., Rajpar, M. N., Majeed, A., & Ditta, A	Applied Ecology & Environmental Research

Supplementary Material. Table: S2: Details of the variables/predictors used in this study. The environmental variables used in modelling potential habitats for demoiselle crane are denoted in bold text

Environmental variables and abbreviations	Abbreviation	Unit	Source
annual mean temperature	Bio1	Degrees Celsius	https://www.worldclim.org/data/worldclim21.html
mean diurnal range (mean of monthly [max temp—min temp])	Bio2	Degrees Celsius	
isothermally (Bio2/Bio7) (*100)	Bio3	Percentage	
temperature seasonality (standard deviation *100)	Bio4	Degrees Celsius	
maximum temperature of warmest month	Bio5	Degrees Celsius	
minimum temperature of coldest month	Bio6	Degrees Celsius	
temperature annual range (Bio5-Bio6)	Bio7	Degrees Celsius	
mean temperature of wettest quarter	Bio8	Degrees Celsius	
mean temperature of driest quarter	Bio9	Degrees Celsius	
mean temperature of warmest quarter	Bio10	Degrees Celsius	
mean temperature of coldest quarter	Bio11	Degrees Celsius	
annual precipitation	Bio12	Millimeters	
precipitation of wettest month	Bio13	Millimeters	
precipitation of driest Month	Bio14	Millimeters	
precipitation seasonality (coefficient of variation)	Bio15	Fraction	
precipitation of wettest quarter	Bio16	Millimeters	
precipitation of driest quarter	Bio17	Millimeters	

precipitation of warmest quarter	Bio18	Millimeters	
precipitation of coldest quarter	Bio19	Millimeters	
human population density of Pakistan	pop		WorldPop,https://www.worldpop.org/doi/10.5258/SOTON/WP00674
elevation above sea level	DEM	Meter	NASA (SRTM)
slope of the area	Slope	Meter	created from SRTM 90m DEM
Road's density	Road		line Density tool in ArcGIS 10.8
digital soil map of the world	Soil		FAO, 2003
Land cover	lulc		USGS: http://edcsns17.cr.usgs.gov/glcc
normalized difference vegetation index	NDVI		USGS: http://edcsns17.cr.usgs.gov/glcc
Terrain Ruggedness Index	rugged		From GIS

Supplementary Material. Figure: S3: Correlation among the 8 variables retained based on the Jackknife test and contribution values

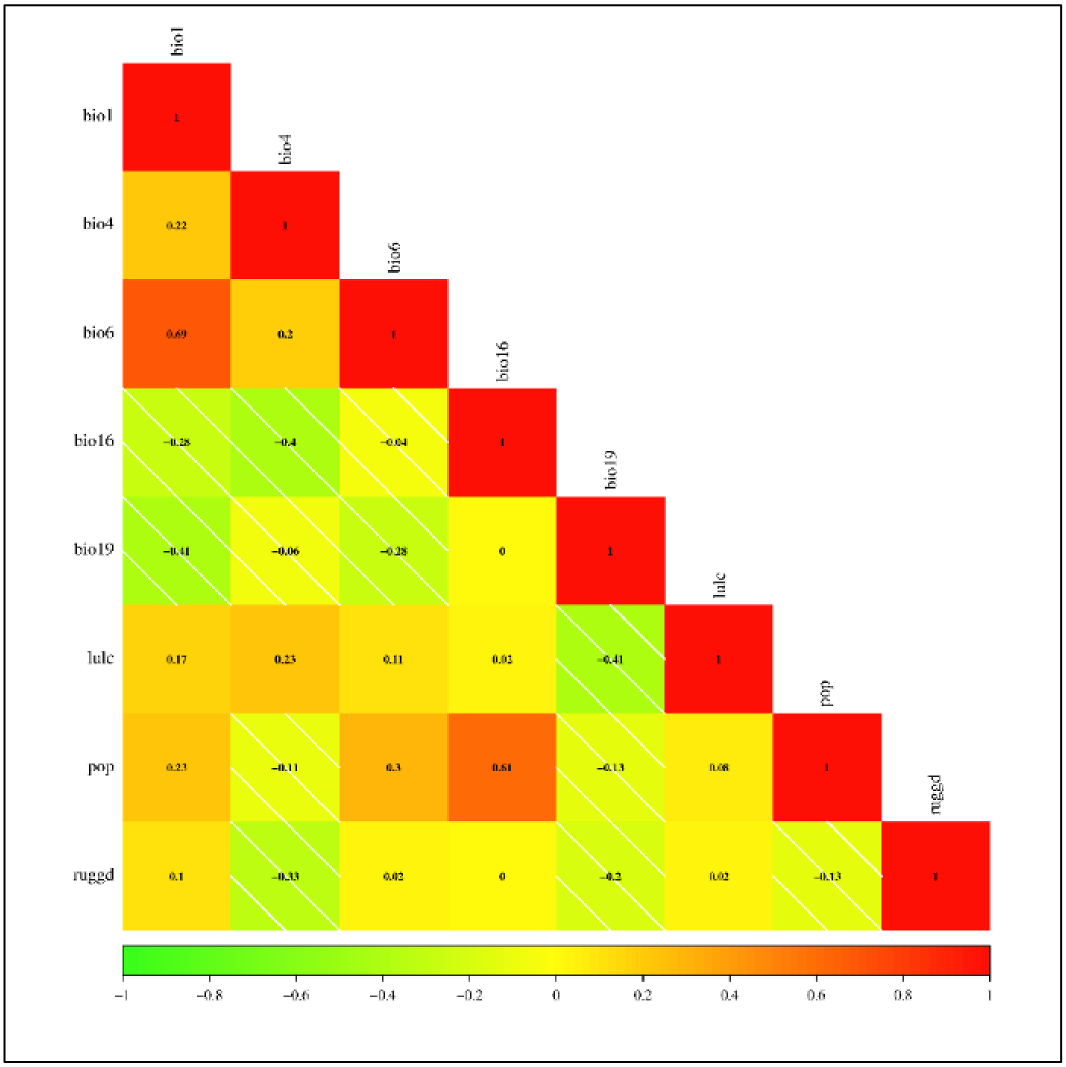

Supplementary Material. Figure: S4: Demoiselle crane habitat suitability (selection) response curves for top four highly contributing predictor variables (Red curves (lines) exhibit the mean response of the ten replicates (runs) of MaxEnt model, while the blue shades indicate mean +/− standard deviation. The range of predictor variable is presented on the X-axis, while habitat suitability index (logistic output) is given on the Y-axis)

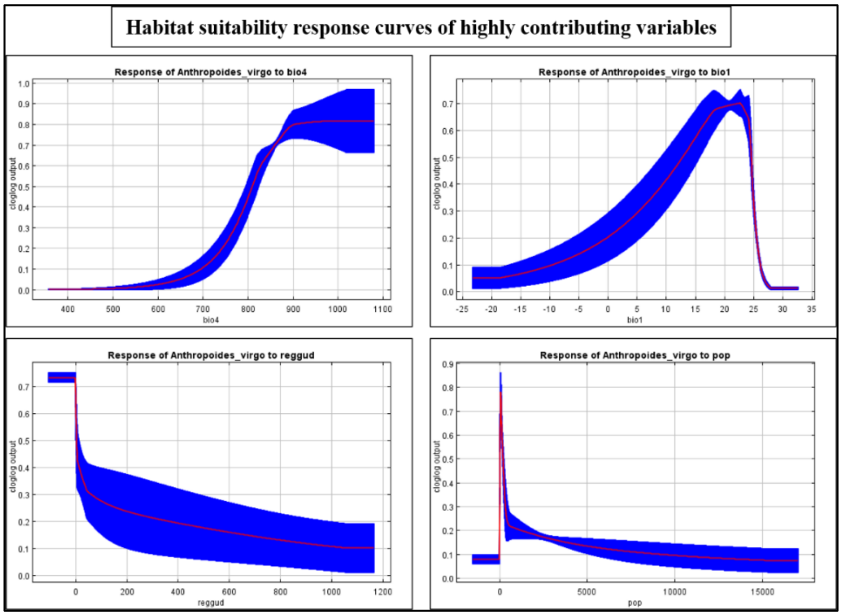

Supplementary Material. Figure: S5: Jackknife test of the regularized training gain of predictor variables tested in the demoiselle crane habitat suitability

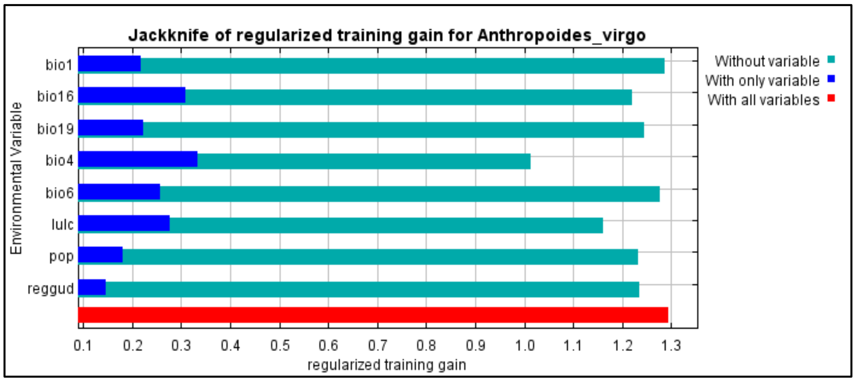

Supplementary Material. Figure: S6: Maps illustrating multivariate environmental similarity surface (MESS) approach for demoiselle cranes under the year 2050 Representative Concentration Pathway (RCP4.5) and (RCP8.5) for different Global Circulation Models. Negative values indicate novel climate in the MESS map across the range.

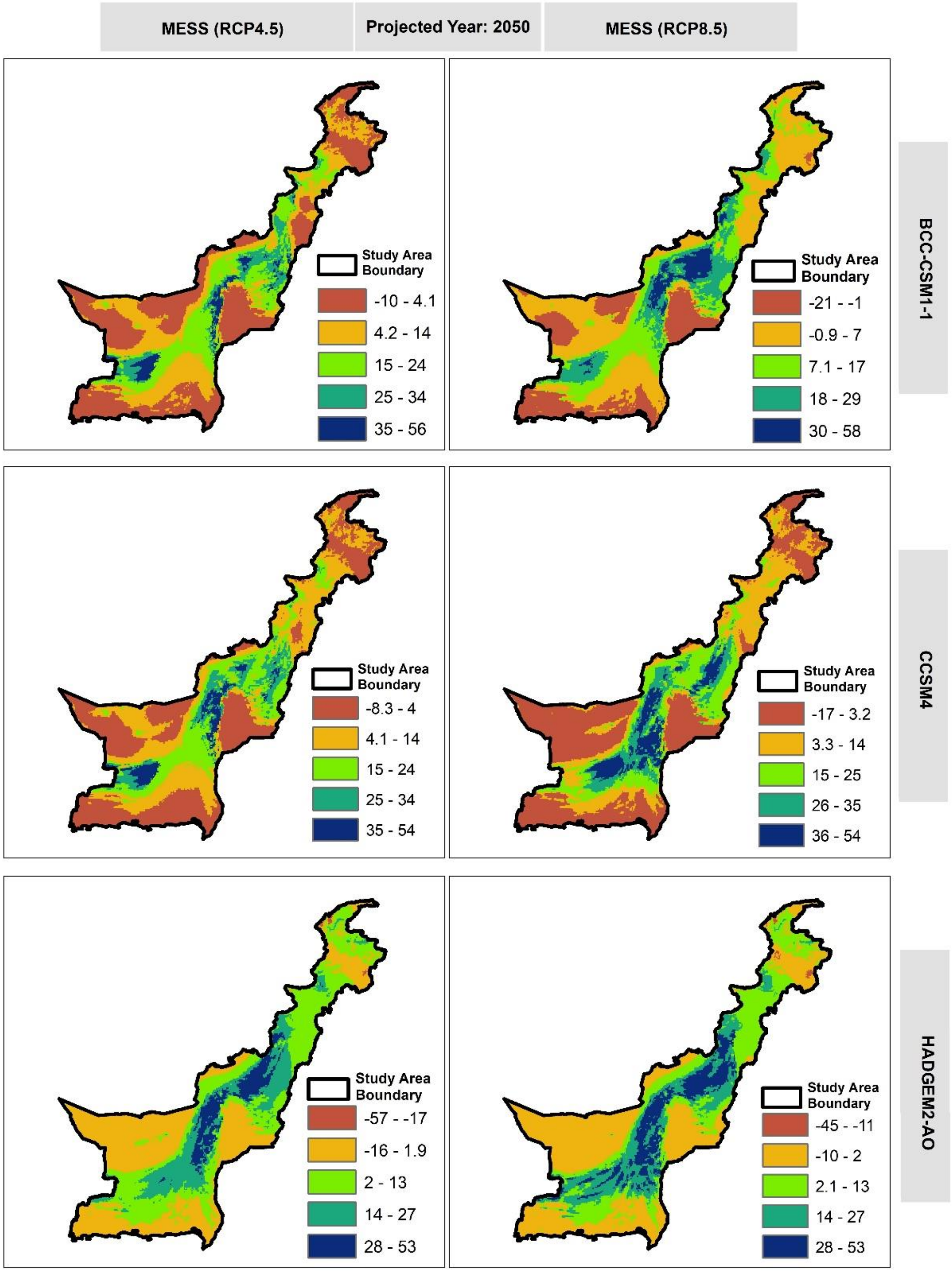

Supplementary Material. Figure: S7: Maps illustrating multivariate environmental similarity surface (MESS) approach for demoiselle cranes under the year 2050 Representative

Concentration Pathway (RCP4.5) and (RCP8.5) for different Global Circulation Models. Negative values indicate novel climate in the MESS map across the range.

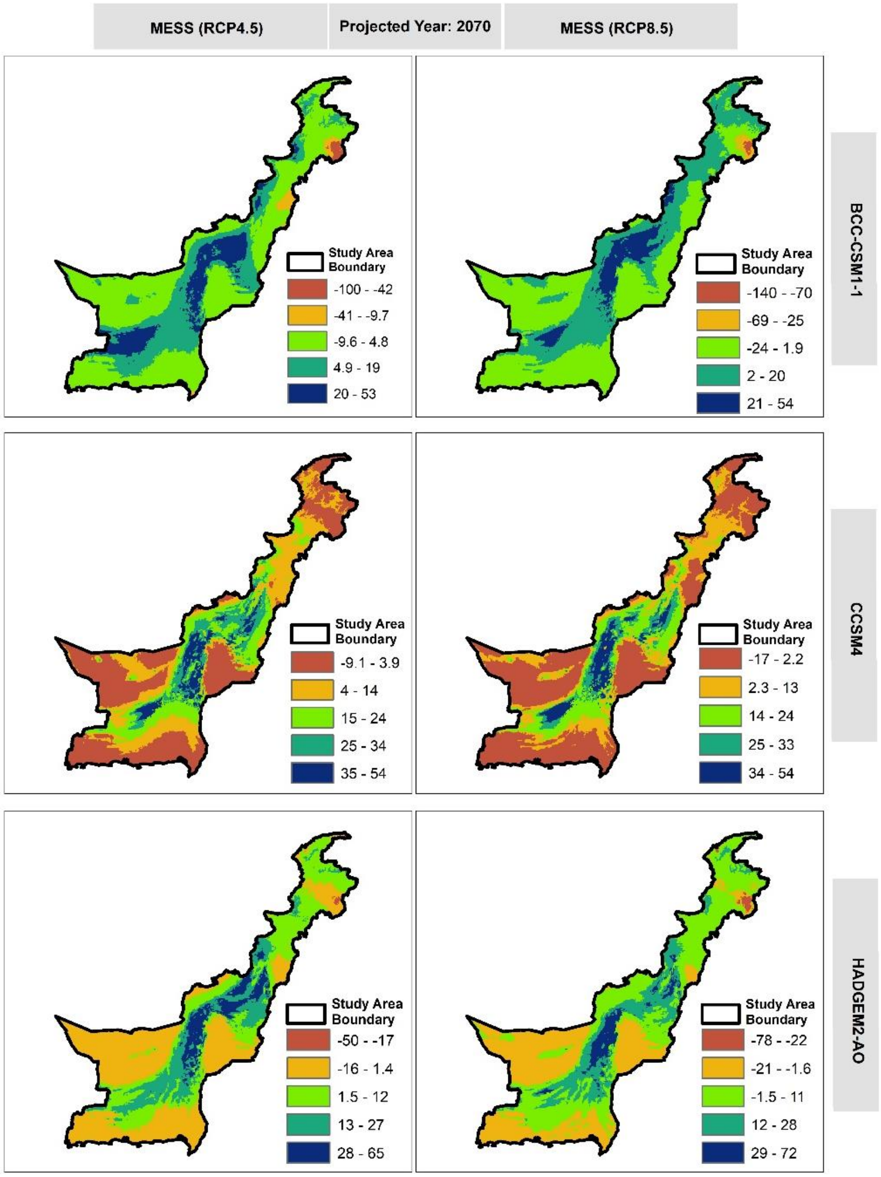

Supplementary Material. Figure: S8: Projection of human population in Pakistan (sources UN)

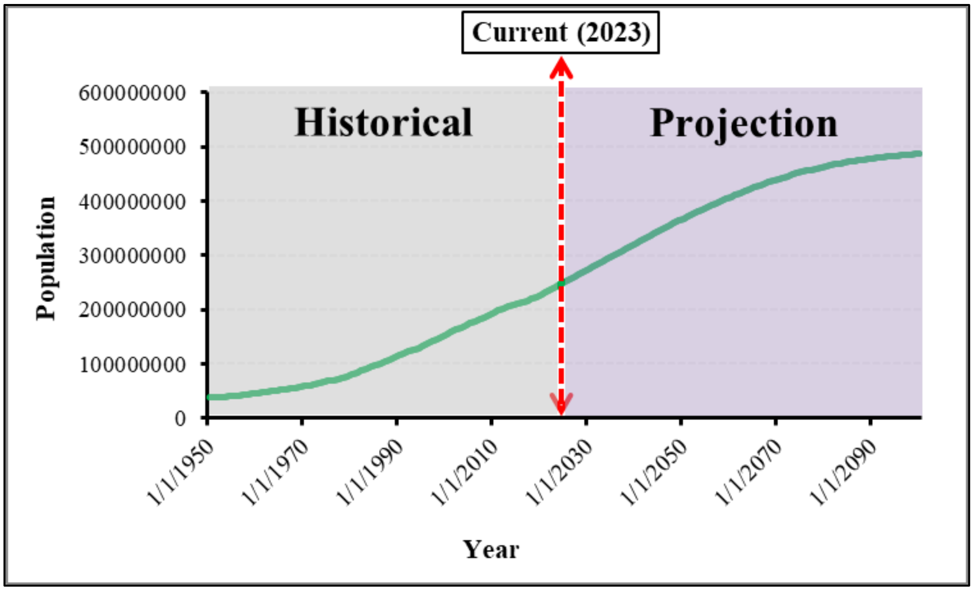

Supplementary Material. Table: S9: Categorization and quantification of Demoiselle Crane habitat (km^2) under current and future climate change

Future Projection			Habitat Categories				
		**Scenario**	Un Suitable	Less Suitable	Moderately Suitable	Highly Suitable	**Total**
Model	Year	Current	267,539	28,865	134,068	27,911	458,383
		RCP4.5	402,917	17,983	23,732	13,751	458,383
	2050	RCP8.5	421,038	18,101	10,122	9,122	458,383
		RCP4.5	399,886	21,568	25,787	11,142	458,383
BCC-CSM1-1	2070	RCP8.5	430,525	9,983	9,983	7,892	458,383
		RCP4.5	317,739	43,873	89,879	6,892	458,383
	2050	RCP8.5	409,880	17,081	21,585	9,837	458,383
		RCP4.5	425,674	10,481	14,197	8,031	458,383
CCSM4	2070	RCP8.5	440,107	8,017	6,046	4,213	458,383
		RCP4.5	371,063	19,938	35,989	31,393	458,383
	2050	RCP8.5	359,904	16,997	46,993	34,489	458,383
		RCP4.5	385,984	11,929	32,881	27,589	458,383
HADGEM2-AO	2070	RCP8.5	398,787	19,345	22,622	17,631	458,383
